# Understanding Inborn Errors of Metabolism through Metabolomics

**DOI:** 10.3390/metabo12050398

**Published:** 2022-04-27

**Authors:** Karen Driesen, Peter Witters

**Affiliations:** 1 Metabolomics Expertise Center, Center for Cancer Biology, VIB Center for Cancer Biology, 3000 Leuven, Belgium; karen.driesen@kuleuven.be; 2 Metabolomics Expertise Center, Department of Oncology, Katholieke Universiteit Leuven, 3000 Leuven, Belgium; 3 Department of Development and Regeneration, Katholieke Universiteit Leuven, 3000 Leuven, Belgium; 4 Center for Metabolic Diseases, Department of Paediatrics, University Hospitals Leuven, 3000 Leuven, Belgium

**Keywords:** inborn errors of metabolism, metabolomics, stable isotopes

## Abstract

Inborn errors of metabolism (IEMs) are rare diseases caused by a defect in a single enzyme, co-factor, or transport protein. For most IEMs, no effective treatment is available and the exact disease mechanism is unknown. The application of metabolomics and, more specifically, tracer metabolomics in IEM research can help to elucidate these disease mechanisms and hence direct novel therapeutic interventions. In this review, we will describe the different approaches to metabolomics in IEM research. We will discuss the strengths and weaknesses of the different sample types that can be used (biofluids, tissues or cells from model organisms; modified cell lines; and patient fibroblasts) and when each of them is appropriate to use.

## 1. Introduction

Metabolism is the sum of all chemical reactions that occur in cells, organs, or organisms. The resulting set of low molecular weight intermediates (metabolites) is analyzed in metabolic studies called ‘metabolomics’ [1]. Metabolomics is one of the latest additions in the ‘omics’ field and compared to genomics, transcriptomics, and proteomics, the study of the metabolome is the closest to the actual phenotype [2]. Therefore, metabolic alterations are a very interesting read-out for both genomic and environmental effects. It can provide information on disease mechanisms and can play a role in diagnostics and therapeutics [3].

Metabolite detection in metabolomics was originally achieved using proton or carbon nuclear magnetic resonance (^1^H- or ^13^C-NMR) spectroscopy [4], but improvements in the mass accuracy of mass spectrometers made mass spectrometry (MS) the preferred method for metabolite analysis [1,5]. NMR spectroscopy has some advantages over MS, such as ease of sample preparation, precise structure determination, and its inherently quantitative nature [5]. Nevertheless, NMR has a lower sensitivity (in the μM range) and can only detect a limited number of metabolites simultaneously [5]. MS allows for the detection of metabolites in the nM range and, when used with liquid chromatography (LC) or gas chromatography (GC), an additional level of separation of metabolites is obtained based on, e.g., polarity (LC) or volatility (GC) [1]. Additionally, tandem mass spectrometry (MS/MS) can help with the identification of metabolites by fragment analysis [6]. 

MS-based metabolomics is normally performed in three steps: sample preparation, data acquisition, and data interpretation [7]. All of these steps are optimized based on the experimental setup. There are two different approaches to metabolomics: untargeted and targeted metabolomics. In untargeted metabolomics, all metabolites extracted from a sample are analyzed. The major advantage of this approach is that it allows for unbiased detection of unexpected metabolic perturbations, which is mainly of importance in biomarker discovery [8]. However, a large number of returned features remain unannotated after a search against metabolic databases [9]. In addition, there is a bias for highly abundant metabolites and no extraction protocol is appropriate for every type of metabolite. Targeted metabolomics, on the other hand, relies on measuring a predefined set of metabolites [10]. These metabolites are chosen based on a priori information on metabolic perturbations and the pathways that can be affected by this. Since the metabolites of interest are known, the extraction protocol can be optimized accordingly. With the use of internal standards, targeted metabolomics measurements can even be (semi-)quantitative [10]. 

One of the most commonly encountered difficulties in metabolomics is that the results can be misleading since they only indicate (relative) concentrations of metabolites. The concentration of metabolites alone cannot give conclusive evidence for pathway activity; even if a certain metabolite is, e.g., significantly downregulated, it can be due to either decreased synthesis or increased breakdown. This is where the power of tracer metabolomics lies: in tracer metabolomics, cells, animals, or even patients are fed with stable isotope-enriched nutrients, most commonly carbon- (^13^C) nitrogen- (^15^N), hydrogen- (^2^H), or oxygen-based (^18^O). The flow of these nutrients can then be followed throughout all connected pathways. A distinction can be made between flux analysis and steady-state metabolomics [8]. In flux analysis, the system still has to reach isotopic equilibrium and is therefore suited to follow dynamic labeling [11]. Flux analysis is frequently used in in vivo research. In steady-state metabolomics, the stable isotope distribution has reached equilibrium which gives information on relative pathway activities and the contribution of a certain nutrient in the production of the metabolites of interest [3]. This extra level of information is invaluable for metabolic pathway activity and subsequent proper disease mechanism elucidation. 

A disease class that can clearly benefit from metabolomics research is inborn errors of metabolism (IEM). IEMs are characterized by a deficient activity of enzymes, co-factors, or transport proteins related to biochemical pathways [12], thereby affecting one or more metabolic reactions. Most IEMs are transmitted as autosomal or X-linked recessive diseases [13]. In 1908, Sir Archibald Garrod was the first who coined the term ‘inborn errors of metabolism’ to describe four disorders affecting normal metabolism [14]. Since then, more and more metabolic diseases have been discovered and currently, over 1450 IEMs are known [15]. Although the individual disorders are rare, the global prevalence of all IEMs is estimated to be 50.9 in 100,000 [16] and, regionally, cumulative incidences of up to 1 in 800 have been reported [17].

IEMs can affect any organ at any age and most patients present with variable clinical presentations and often non-specific symptoms, such as failure to thrive, hypotonia, renal and hepatic diseases, and intellectual disability [18,19]. The absence of pathognomonic signs and symptoms makes it difficult for physicians to decide when to test for IEMs and to perform the correct laboratory testing. Additionally, there is a low incidence of most disorders and thus a low pre-test probability. 

IEMs have led to the development of newborn screening (NBS) programs. Although NBS already started in the 1930s, its breakthrough came in 1963 when Robert Guthrie designed a fast and simple test for phenylketonuria using dried blood spots (DBS) taken from the heels of neonates [20]. The next boost for NBS came when tandem mass spectrometry (MS/MS) was introduced [21]. This allowed a much larger number of disorders to be detected from a single blood spot [21]. In 1986, Wilson and Jungner published ten ‘rules’ for the inclusion of conditions in NBS programs, such as prevalence and treatability [22]. These rules still form the basis of the current guidelines, although they have been adapted since, in response to the major advances in molecular testing [23]. Every country can make its own selection of what disorders are included in NBS [23,24]. This leads to major regional differences between which IEMs are included. Importantly, NBS is not a diagnostic tool, so abnormal newborn screens still have to be diagnostically confirmed or excluded (to eliminate false positives) [24]. Also, it is important to note that a normal NBS does not exclude the possibility of an IEM as only a small subset of IEMs are included in NBS programs.

The diagnosis of IEMs is different for each disorder, but it is generally a combination of biochemical, molecular, or enzymatic testing [25]. Biochemical tests are performed on blood and urine samples often by mass spectrometry and rely on the detection of high levels of metabolites that are known biomarkers for a certain enzymatic block. If the biochemical tests are indicative of an IEM, the genes of interest can be sequenced by Sanger or next-generation sequencing to confirm the diagnosis. For some disorders, the enzymatic activity is a good read-out for disease, but for many IEMs enzymatic testing is not available and residual enzyme activity can be indicative of disease severity, but the correlation is not always present. Lately, there has been an increase in the use of untargeted genomic testing in the search for the correct diagnosis [26]. Biochemical testing remains essential since it is often faster, it can give information about the disease severity, and it can assist in the interpretation of variants of unknown significance that are often found in DNA sequencing [26].

For diagnostic tests, clinicians focus first on IEMs that have an effective treatment available, so the patients can maximally benefit from an altered disease course. For most IEMs, no effective therapy is available, but for the treatable ones, the treatments rely on overcoming the metabolic defect [25]. This can be achieved by supplementing deficient products or preventing toxic build-ups, for instance by nutritional therapy, by increasing the disposal of toxic intermediates, by activating alternative pathways to metabolize them, or by replacing the defective enzyme with a functional one (by enzyme replacement therapy). It is clear that most of these treatments are tailored for the metabolic defect in a simplified way, i.e., looking at one specific metabolic block without taking into account secondary cellular adaptations to this metabolic dysregulation. 

Overall, there is still a substantial knowledge gap on how a single enzyme deficiency affects multiple pathways and how this can be overcome in the form of therapy. Metabolomics can provide information on the perturbations in multiple pathways and thus is more appropriate than focusing on a single enzyme. Therefore, this review will give an overview of the use of metabolomics in IEM research in patients, relevant model organisms, and cell models, and how using stable isotopes can help elucidate the mechanisms at hand. This will give researchers an idea of how to implement (tracer) metabolomics in their research. The list of papers included is not exhaustive, but merely an illustration of the different approaches that can be used in metabolomics. An overview of all the included papers and their key findings is given in Table A1.

## 2. In Vivo and Ex Vivo Metabolomics

**Figure 1:**
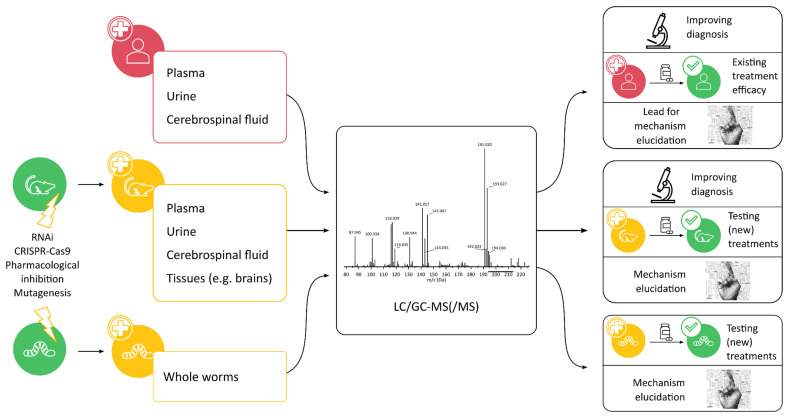
Overview of the different biological models for the study of IEM and the subsequent information that can be obtained from metabolomics studies.

In vivo and in vitro metabolomics rely on very different sample types, sample preparation, experimental setup, and the resulting data. Since these are the pillars of metabolomics research, a distinction will be made between IEM research in vivo and in vitro. An overview of the information that can be extracted from each in vivo model included in this section can be found in Figure 1. 

### 2.1. Analysis of Patient Samples

Metabolomics studies performed directly on patients are mostly relevant for biomarker discovery and, although they can be carried out, are not as well-suited for pathomechanism elucidation. This is because it is harder to obtain all of the necessary information from blood, urine, and cerebrospinal fluid. However, research on human samples is very valuable because it can give information on how to achieve a more efficient diagnosis, on disease progression, and on treatment efficacy or compliance. Additionally, pathway analysis after (un) targeted metabolomics can identify the metabolic pathways that are most severely affected in the disease, although it is difficult to understand how much these contribute to pathophysiology. Providing patients with stable isotopes (e.g., oral or intravenous administration) and measuring where these are incorporated may help to solve these questions. Another use of stable isotopes in vivo is to check isotope dilution to quantify the fluxes to certain pathways in real time, but this is beyond the scope of this review and has been described extensively elsewhere [11]. 

#### 2.1.1. Biomarker Discovery for Diagnosis

Biomarker discovery for improving diagnosis can be of use, e.g., in NBS programs or as a diagnostic marker. It is mostly performed using untargeted metabolomics on human plasma since this allows for the largest set of results. Detailed reviews specifically on this topic have already been published [27], so this will not be discussed here.

#### 2.1.2. Treatment Efficacy

In this type of study, patients who are receiving some treatment are checked for clinical biomarkers for disease severity related to their respective IEM. Another area of research in this is finding suitable biomarkers for monitoring treatment. 

For the latter, a study was performed on plasma, urine, and cerebrospinal fluid (CSF) samples of patients with a glucose transporter type 1 (GLUT1) deficiency [28]. This can lead to microcephaly, seizures, developmental delay, and movement disorders, aggravated by fasting, as the brain is partly deprived of its main fuel, glucose. The CSF was taken from patients before treatment started and the plasma and urine samples were obtained while on the ketogenic diet (KD). Ketone bodies provide energy to the brain independent of the GLUT1 dysfunction. The authors used an untargeted metabolomics approach to find what metabolites were significantly altered in the disease state compared to controls and in patients before and during treatment. They found that the carbohydrate and lipid metabolism were affected the most in the KD patients and the amino acid profiles also showed large perturbations. Additionally, they found severely depleted levels of free carnitine on KD treatment, which can affect tricarboxylic acid (TCA) cycle intermediates. Therefore, they propose supplementation with L-carnitine for patients on a ketogenic diet. 

Ney et al. focused on the bioavailability of tyrosine and tryptophan from two different types of amino acid supplementation for a low phenylalanine diet: amino acid medical food (AA-MF) and glycomacropeptide medical food (GMP-MF) in phenylketonuria (PKU) [29]. PKU results in the accumulation of phenylalanine in the blood and brain. If left untreated, patients develop severe intellectual disabilities, as well as eczema, hyperactivity, and recurrent seizures [30]. Based on previous evidence [31], the authors postulate that deficiencies of tyrosine and tryptophan are the basis of neurocognitive symptoms in PKU; tyrosine and tryptophan are precursors of dopamine and serotonin, respectively, which are both important neurotransmitters. To test their hypothesis, they performed an untargeted metabolomics analysis on plasma and urine samples from PKU patients on either of the treatments. In patients receiving AA-MF, the authors demonstrate that, although the dietary intake of tyrosine is higher, the bioavailability is lower compared to tyrosine in GMP-MF. This was proven to be caused by intestinal degradation to potentially harmful intermediates. Secondly, a higher intake of leucine, isoleucine, and threonine from GMP-MF competes with phenylalanine for transport into the brain, thereby reducing phenylalanine levels in the brain and improving the bioavailability of tyrosine and tryptophan for neurotransmitter synthesis. Finally, they found that patients using AA-MF metabolized a larger proportion of tryptophan through the kynurenine pathway than through serotonin synthesis, resulting in a build-up of quinolinic acid. Increased levels of quinolinic acid result in oxidative stress and neural loss. From these results, it appears as though GMP-MF is a more appropriate amino acid supplement for PKU patients. 

Similar studies were performed on urea cycle disorders [32], glycogen storage disease [33], fatty acid disorders [34], and mitochondrial dysfunction [35] (see Table A1). 

Interestingly, tracer metabolomics has been used to assess treatment efficacy by probing the rate of ureagenesis in patients with two urea cycle disorders before and after treatment with N-carbamyl-L-glutamate (NCG) [36,37]. In these studies, ureagenesis was used as a critical outcome measure to assess the efficacy of this new treatment for urea cycle disorders. The patients were given oral doses of 1-^13^C-acetate and the levels of ^13^C-urea were sampled at different time points. For patients with ornithine transcarbamylase deficiency and N-acetylglutamate synthase deficiency, the production of ^13^C-urea before treatment was low, but after three days of supplementation with NCG, the levels increased significantly. Also, blood urea nitrogen increased after treatment while glutamine and alanine levels decreased. This shows an improvement in disease severity due to NCG supplementation. 

#### 2.1.3. Mechanism Elucidation 

The source of hyperlipidemia in two patients with glycogen storage disease Ia (GSD Ia) was investigated by Bandsma et al. [38]. GSD Ia patients mostly suffer from recurrent hypoglycemia and liver disease. The disease is caused by a deficiency of glucose-6-phosphatase, which causes primary abnormalities in glucose metabolism. To find the relation between disturbed glucose metabolism and hyperlipidemia, the patients and six healthy volunteers were infused with 1-^13^C-acetate. The incorporation of stable isotopes in acetyl-CoA was lower in the patients compared to the controls, which is indicative of increased endogenous production of acetyl-CoA from glucose. This higher glycolytic flux is also highlighted by higher levels of lactate. In turn, these higher acetyl-CoA pools can stimulate acetyl-CoA carboxylase and thus increase lipogenesis. 

Tebani et al. published a series of papers in which they carried out untargeted metabolomics on the urine samples of patients suffering from three lysosomal storage diseases, mucopolysaccharidosis (MPS) I, III and VI [39,40,41]. MPS patients suffer from a progressive multisystemic disease with intellectual disability, impaired motor function, hepatosplenomegaly, cardiomyopathy, and orthopedic, vision and hearing impairment. This analysis gave similar results for all MPS types; all of them showed major dysregulations in amino acid catabolism and fatty acid pathways. Quantification of the amino acids and subsequent pathway analysis led them to find the specific pathway that was the most affected, which was arginine–proline metabolism. Since this is closely linked with the urea cycle, this was upregulated as well. This approach is most interesting to find unexpected alterations in unrelated pathways, which can be a lead for full mechanism elucidation. 

### 2.2. Model Organisms to Study IEMs

Model organisms are indispensable for metabolomics research because of the difficulties inherent to studying patients directly. These difficulties include the limited control over environmental influences and the possibility of other genetic defects that can cause alterations to the metabolome. Additionally, testing of new therapies and the knockout of different genes is easy to achieve in a model organism. Reed et al. published an extensive overview of the organisms available for metabolomics research and their strengths and weaknesses [42]. In short, organisms such as mice, worms, flies, yeast, bacteria, or zebrafish are widely used in metabolomics research. These organisms can be genetically altered quite efficiently and can therefore serve as suitable model organisms for IEMs. The mutations can be made by chemical mutagenesis, RNAi, CRISPR-Cas9, or by pharmacological inhibition. The whole organism, or organs that are important in the disease but hard to examine in patients (e.g., brain), can be analyzed. For mice, plasma and urine samples can be analyzed as well for direct comparison with humans. We will only discuss research based on mice and worms in the following section since these are the model organisms that are most frequently used for metabolomics research in IEMs. 

#### 2.2.1. Mice (*Mus musculus*)

There are several papers handling the use of mice for biomarker discovery using untargeted metabolomics, either in tissues or plasma, but this is outside the scope of this review and will not be discussed in detail [43]. 

The metabolic origins of neurological dysfunction in PKU were examined by Lu et al. in cerebral cortices from homozygous Pah^enu2−/−^ mice (PKU mice) compared with wild-type mice (WT mice) [44]. First, a metabolomics screening revealed that 35 metabolic compounds were significantly altered in the brain tissue of PKU mice compared to WT. These compounds were further analyzed by pathway analysis. This revealed that several pathways were significantly altered in PKU mice and offered several possible explanations for the neurological damage in PKU: (i) elevated levels of phenylalanine and its metabolites cause oxidative stress and subsequently inhibit cell proliferation, (ii) lower levels of branched-chain amino acids interferes with the biosynthesis of proteins, brain metabolites, or neurotransmitters, (iii) the enrichment of aspartate, gamma-aminobutyric acid, and glutamate may interfere with important neurotransmission functions, (iv) elevated purine metabolism intermediates disturb their neuroprotective function. 

The forebrains of Glut1 deficient (G1D) mice were examined metabolically by Marin-Valencia et al. [45]. They found lowered acetyl-CoA levels but normal abundances of TCA cycle intermediates. This suggests either alternative fueling of the TCA cycle or an increased flux of acetyl-CoA into the TCA cycle. Secondly, the brains of the G1D mice had lower levels of free fatty acids, while cholesterol and triglyceride levels were normal. 

Hijmans et al. used tracers to check the flux through pathways they expected to be affected in a mouse model of GSD Ia [46]. Although the mice only had 5% residual glucose-6-phosphatase activity in vivo, they still had 30% residual endogenous glucose production compared to wild-type controls. Therefore, they set out to find where this glucose was coming from and found that treatment of hepatocytes of the KO mice with an inhibitor for two hepatic α-glucosidases almost completely stopped the production of free glucose. This led them to conclude that cytosolic glycogen degradation was responsible for the production of free glucose. 

In a mouse model of alkaptonuria (AKU), the urinary profiles of homozygous AKU mice were compared to those of heterozygous healthy mice [47]. This disease is characterized by urine that turns black when exposed to air, discoloration in the eyes, ears and skin, early-onset osteoarthritis, and kidney and prostate stones. This analysis was carried out with both untargeted and targeted metabolomics. Although the untargeted metabolomics only found significant differences in eleven metabolites that yielded no matches in a search against a database, the targeted metabolomics showed the expected perturbations in homogentisic acid (HGA) and its biotransformation products. In a second experiment, the levels of these metabolites were checked in urine samples of the AKU mice and patients on nitisinone treatment, which is the standard of care in AKU. This showed significant decreases in HGA and its products in mice and patients. To find which biotransformation products are predominantly formed, the AKU mice were infused with ^13^C_6_-HGA and the incorporation in the other upregulated products was followed. A major upregulation of phase II biotransformations was found, primarily in glucuronidation and sulfation reactions. This is probably an attempt to detoxify HGA before it oxidizes to highly reactive benzoquinone and free radicals. Since this detoxification seems so important, further activation of the enzymes in phase II biotransformations could serve as a new therapeutic modality. 

Mice have historically been a favored model organism for IEM research [48], owing to their efficient genetic alteration. Since plasma, urine, and cerebrospinal fluid, although in small quantities, can be extracted from mice, they can provide a complete comparison to human data. This can be used in conjunction with additional information from, i.e., organs to find the mechanisms at play. Although mice are genetically similar to humans, not all symptoms associated with a certain disease in patients are present in mutant mice. Finally, the high costs and ethical constraints associated with experiments in mice have led researchers to use alternatives such as worms. 

#### 2.2.2. Worms (*Caenorhabditis elegans*)

Falk and co-workers published a series of metabolomic studies on the use of *C. elegans* with different mitochondrial disorders [47,48,49]. Mitochondrial disorders are a large group of diseases, but common symptoms include stunted growth, low muscle tone, intellectual disability, and impaired vision and hearing. In 2008, they compared complex I, II or III, and coenzyme Q mutant worms in a more explorative study of the amino acid pools [49] and found decreased levels of phenylalanine and arginine and increased levels of the branched-chain amino acids and alanine. The decrease was attributed to increased amino acid degradation to fuel the oxidative phosphorylation (OXPHOS) while the increases are explained to originate from the decreased oxidation of pyruvate and α-ketoglutarate. In a follow-up study, Vergano et al. included four more models, one with a defect in the TCA cycle, one with a defect in pyruvate metabolism, and two with defects in the nutrient-sensing signaling network [50]. The worms were fed 1,6-^13^C_2_-glucose or U-^13^C-glucose and the isotopic incorporation into glycolytic and TCA intermediates was tracked. For the ^13^C_2_-labeled glucose, increases in M1 lactate were found as well as decreased M1 glutamate and aspartate. This can be attributed to a higher glycolytic flux to lactate, which can be caused by a high NADH/NAD^+^ redox ratio. Also, a decrease in TCA cycle activity can be concluded. The results are different for all models, but more information about the most affected pathway can be obtained because it can be compared to the knockout of the other enzymes. In a different model of secondary mitochondrial dysfunction, propionic acidemia, no significant difference in amino acid metabolism was observed except for cysteine [51]. To find the mechanisms at play, the worms were incubated with 1,6-^13^C_2_-glucose to check the pathway activities of glycolysis, pyruvate metabolism, and the TCA cycle. They found no decrease in flux through glycolysis and pyruvate, but an increased M1 citrate pool and almost non-existent M1 malate pool indicate that the distal TCA cycle is impaired in this worm model. 

*C. elegans* can be a good model for IEMs since they are relatively cheap to maintain, easy to extract, and can be bought as knock-out worms or be altered with RNAi. Additionally, they have a short generation time and short lifespan and their main route of reproduction is through self-fertilization. This hermaphroditic reproduction leads to low genetic variation [52]. Although not all metabolic pathways are the same as in humans, the most important ones such as glycolysis, the TCA cycle, and PPP are conserved in this animal model. The main limitation of this model is that not all genes in humans find their homologues in *C. elegans*. This means that not all IEMs can be reproduced by RNAi and it can be challenging to translate data found in *C. elegans* to humans. Moreover, *C. elegans* are not very appropriate for biomarker discovery since analogues of plasma or urine cannot be easily extracted from these organisms.

## 3. In Vitro Metabolomics

**Figure 2:**
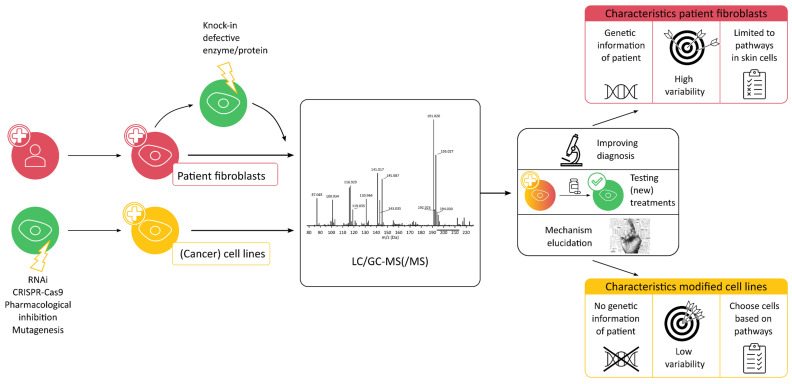
Overview of the information that can be obtained from both in vitro models in metabolomics studies of IEM.

Although model organisms already have increased control over variables compared to human samples, in vitro experiments are even more suited for minimizing variability between samples. Both cell lines and primary cells taken from patients are easy to work with, comparatively cheap, and the environmental factors are easier to control [53]. With in vitro techniques, researchers can focus on the effects in a single tissue, which simplifies the data interpretation, while it is highly complementary to the results in the whole system. Recently, organoids have gained attention as a 3D cell culture technique that can mimic organs but is grown from induced pluripotent stem cells. They could be interesting for metabolomics research in IEMs and have been used as a model of an intestinal disorder [54], but no papers have been published yet, in which organoids were used for metabolomics. Figure 2 summarizes the limitations and advantages of both types of cells discussed in the following sections. 

### 3.1. Modified Cell Lines

The cell lines that are modified are mostly immortal cancer cell lines that are commercially available. Similar to animal models, the genetic modifications can be carried out by RNAi, zinc-finger nucleases, CRISPR-cas9, or pharmacological inhibition [55]. 

In an effort to study mitochondrial dysfunction, HEK-293-derived cells were transfected with a mutant DNA polymerase gamma to halt the replication of mitochondrial DNA (mtDNA) [56]. Metabolic profiling revealed serine as the most upregulated metabolite compared to wild types, which was confirmed in the treatment of the wild-type cells with EtBr, which also depletes mtDNA. To find out if the high levels of serine were caused by increased biosynthesis or decreased breakdown, the authors did a ^13^C-glucose tracer experiment. The results showed a combination of both; the cells produce more serine from glucose and take up less serine. This implies less consumption of serine, which results in the reduced production of folate. This shows an impaired one-carbon metabolism in cells with mitochondrial dysfunction. 

In 2018, Gaude et al. replicated mitochondrial dysfunction in human osteosarcoma cells with mitochondrially targeted zinc-finger nucleases to generate cell lines with varying levels of mtDNA mutation [57]. The induced mutation affects a subunit of ATP synthase. They demonstrated an increase in reductive carboxylation regulated by malate dehydrogenase 1 (MDH1). The conversion of oxaloacetate to malate recycles NADH, which can be immediately used by GAPDH in glycolysis. There is an increased need for glycolysis for ATP generation in mitochondrial dysfunctions. The malate generated by MDH1 is normally used in the malate–aspartate shuttle (MAS), but these cells undergo rewiring of the MAS, as proven by the low levels of aspartate. 

The major advantage of using (commercially available) cell lines is that these do not meet the same strict ethical considerations as human or animal samples and do not require gender- or age-matching for control conditions [53]. Rather, an immediate control is available in the form of the unmodified cell line. Additionally, these immortalized cell lines provide an unlimited and homogeneous population, but keeping the cells for too many passages may result in a genetic drift [58]. Another downside of genetically altered cell lines is that these cells do not have the whole genetic constitution of patients and can therefore be more difficult to compare with whole-organism data. Finally, if the mutation is caused by viral transduction, the viral genes can interact with metabolic pathways, which can result in changes in the metabolome that are unrelated to the IEM itself.

### 3.2. Patient Fibroblasts

Fibroblasts are taken from patients in a skin biopsy, which is often carried out for diagnostic purposes and can later be used for scientific research. An often-used technique is to do a knock-in of the wild-type gene to assess if the observed changes are only associated with the mutation or if there are secondary mutations that also alter the metabolome.

Salomons and coworkers found that combined D-2- and L-2-hydroxyglutaric aciduria is caused by a mutation in SLC25A1, the mitochondrial citrate carrier [59]. The mutation was found using WES on genomic DNA from blood, but from genetic data alone, no conclusion could be made on the pathogenicity of this mutation. Therefore, patient fibroblasts were incubated with U-^13^C-glucose, and the incorporation of the isotopes into citrate was tracked by MS/MS. They found lower levels of M2 citrate in the culture medium, which reflects an impaired citrate efflux. Moreover, the fibroblasts had higher levels of D-2- and L-2-hydroxyglutaric acid (D,L-2-HGA) and normal levels of TCA cycle metabolites downstream of citrate. To confirm these findings, the fibroblasts of one patient were transfected with wild-type SLC25A1 to restore SLC25A1 levels [60]. This resulted in an increased efflux of citrate, as well as a decrease in intracellular D,L-2-HGA, which proves that the mutation is responsible for the pathology in these patients.

Targeted metabolomics was used to assess the metabolic alterations in skin fibroblasts taken from GSD I and III patients [61]. This revealed a decreased activity of glycolysis and more expenditure of TCA cycle metabolites into OXPHOS. This was suggested by lower levels of succinate, the entry point of the TCA cycle into complex II of OXPHOS. The increased energy demand can produce an excess of reactive oxygen species that have to be countered by the action of the antioxidants cysteine and glutathione. Analysis of these compounds revealed a depletion of these redox systems. For a more complete insight into the adjustments of the energy metabolism in GSD patients, one could use stable isotopes to check the incorporation into glycolysis, fatty acid oxidation, amino acids biosynthesis, and cellular energy reserves [61].

Ni et al. studied two different causes of mitochondrial dysfunction in patient fibroblasts: lipoyltransferase 1 (LIPT1) deficiency [62] and glutamyl-tRNA synthetase 2 (EARS2) deficiency [59]. LIPT1 is necessary for the correct functioning of 2-ketoacid dehydrogenase (2KDH), so a deficiency leads to disordered oxidative metabolism. This expectation corresponds with the metabolic profile of the patient fibroblasts: there was an increase in the levels of α-ketoglutarate, as well as in alanine and lactate which is common for a decrease in pyruvate dehydrogenase activity. This activity was indeed found to be lower in the patients, as evidenced by a decreased release of ^14^CO_2_ from ^14^C-pyruvate. A tracer study with U-^13^C-glutamine revealed that the cells used both the oxidative and reductive TCA cycle, but the oxidative cycle was still predominant. The patient cells also showed more incorporation of glutamine into palmitate, whereas the contribution from U-^13^C-glucose was suppressed. This is also associated with an increase in reductive glutamine metabolism. Expression of wild-type LIPT1 in the patient cells restored glutamine oxidation, which proves that the symptoms arise from a mutation in LIPT1. For the second cause of mitochondrial dysfunction, EARS2 deficiency, the fibroblasts also showed abnormalities in the TCA cycle, but on top of that, purine and pyrimidine metabolism were also significantly altered [63]. In this study, no tracers were used, so it is not possible to conclude if the pathways are activated or suppressed.

Finally, congenital disorders of glycosylation (CDGs) are frequently studied in fibroblasts using (tracer) metabolomics. CDGs can present with a large range of symptoms, such as poor growth, intellectual disability, seizures, liver disease, and low muscle tone. Radenkovic et al. examined the origins of galactose in N-glycans in phosphoglucomutase 1 (PGM1) CDG, while simultaneously describing the pathophysiology associated with PGM1-CDG [64]. They sought to find an explanation for how galactose treatment improves the outcome for PGM1-CDG patients by following the fate of U-^13^C-glucose and U-^13^C-galactose in patient fibroblasts. For cells with and without galactose treatment, the pentose phosphate pathway was significantly increased, although galactose does not contribute to this. Interestingly, they revealed that galactose-1-phosphate was severely depleted in PGM1-CDG cells and this was replenished with galactose therapy. Additionally, the depletion of UDP-glucose and UDP-galactose was restored when the cells were given galactose. This suggests that the treatment is effective because it regulates the UDP-hexose (such as UDP-glucose through the Leloir pathway) levels. This was also reflected in the composition of the N-glycans, in which exogenous galactose replaces glucose as a source for galactose.

In an attempt to understand the source of mannose in N-glycans in phosphomannose isomerase (MPI) CDG, GC-MS analysis was performed [65]. Healthy fibroblasts were supplemented with 1,2-^13^C-glucose and 4-^13^C-mannose and the N-glycans were removed and hydrolyzed to their constituting hexoses. The labeling pattern in the hexoses was examined, which showed that only a small amount of exogenous mannose is incorporated into glycans, but the incorporation of mannose is still more efficient than that of glucose. They also found that glycogen, gluconeogenesis, or recycled mannose do not contribute to N-glycans under physiological conditions.

Metabolomics can also be used to confirm whether a certain enzyme defect leads to a CDG. Morava et al. studied patients with a deficiency in phosphoglucomutase 2 like 1 (PGM2L1), an enzyme that catalyzes the formation of glucose-1,6-bisphosphate and other hexose- and pentose-bisphosphates [66]. Since glucose-1,6-bisphosphate is a potent activator of phosphomutases, a decrease in NDP-sugar formation was expected. However, except for a decrease in glucose-1,6-bisphosphate, no differences between patient and control fibroblasts were found. In addition, no glycosylation defect was found, so PGM2L1 deficiency probably does not result in a CDG, except possibly in the brain. However, it is more plausible that PGM2L1 has a regulatory role in sugar metabolism in the brain and, in that way, causes the neurodevelopmental disorder seen in the patients. Another enzyme defect that was examined for glycosylation defects was related to a deficiency in the trafficking protein particle complex subunit 9 (TRAPPC9) [67]. For metabolites directly linked to glycosylation, higher UDP-hexose levels and lower levels of sialic acid were found in the patient cells, which is indicative of a CDG. Upon examining the cells for defects in central carbon metabolism, a diminished ratio of NAD+/NADH was found. Using U-^13^C-glucose, an increase in the upper glycolytic intermediates was found in patient cells, including the triose phosphates which are precursors for triglyceride synthesis. Additionally, more glucose was found to enter the TCA cycle via pyruvate dehydrogenase and the PPP intermediates were increased in the patient cells.

The major advantage of using fibroblasts from patients is that it contains the genetic information from the patient and, in this way, can provide direct information about what is happening inside the patient. The usefulness of fibroblasts is underlined by the conservation of most metabolic pathways in this cell line, but for some more specialized functions, a different cell line should be used. A prime example is gluconeogenesis, which should be studied in hepatic cells. However, these cells are not readily accessible in humans, so either established cell lines (e.g., HepG2) or animal models have to be used as a source of these cells. Another disadvantage of using fibroblasts is that these primary cells can only be used for a limited number of passages [58]. Moreover, the fact that these cells originate from patients causes them to have higher variability, due to sex, age, and environmental factors. What makes it even more challenging is that the control cells have to be obtained by skin biopsy from healthy people.

## 4. Conclusions and Recommendations

Inborn errors of metabolism are monogenic, often untreatable, disorders, in which a single enzyme or protein causes a distinct metabolic signature. Research in to inborn errors of metabolism has already benefitted from metabolomics as a tool to facilitate diagnosis by finding appropriate biomarkers in human samples. Now, metabolomics has emerged as a powerful technique to investigate disease mechanisms in different model systems. These include genetically altered mice or worms and modified cell lines or primary cells taken from patients. Each of these can serve as a suitable model and, by combining different models, the data that can be extracted will be even more informative. The application of stable isotopes in so-called tracer metabolomics can help to further assess the exact mechanisms at play. Based on these mechanisms, new therapies for previously untreatable disorders have been proposed and metabolomic profiling can be used to assess the mode of action and efficacy of the treatment. There is still a substantial knowledge gap in the pathophysiology underlying most inborn errors of metabolism, so the application of metabolomics to these diseases could prove to be invaluable for future disease handling.

A “multi-omics” approach that combines metabolomics with different ‘omics’ such as genomics or transcriptomics can help to complete the picture of the IEM disease profile [68]. This approach allows for the integration of different levels of data and, in this way, provide insights beyond a genotype–phenotype correlation that is often absent in IEMs. Moreover, combining this with clinical insights might help clinicians ameliorate the screening and diagnostic practices that are now in place and thus improve the outcome for patients suffering from IEMs.

## Appendix A

**Table A1:** Overview of the papers included in the review.

Inborn Error of Metabolism	Model System	Induced Mutation	Treatment	Stable Isotopes	Key Findings (Affected Pathways/Metabolites)
Disorders affecting small molecules					
Phenylketonuria [29]	Human plasma and urine	N/A	Amino acid -medical food (AA-MF) vs. Glycomacro-peptide-MF (GMP-MF)	N/A	- Higher bioavailability of tyrosine and tryptophan from GMP-MF than from AA-MF - More metabolization of tryptophan through kynurenine pathway, resulting in oxidative stress - Higher levels of BCAAs in GMP-MF reduce phenylalanine levels in the brain
Phenylketonuria [44]	Mouse brains	Chemical ENU induced mutation to Pah ^enu2+/−^ mice that were interbred	N/A	N/A	- High levels of phenylalanine - Low levels of BCAAs - High levels of aspartic acid, gamma-aminobutyric acid, and glutamate - Purine metabolism more active
Alkaptonuria [47]	Mouse and human urine	Inducible knockout of HGD	Nitisinone	^13^C_6_-homogen-tisic acid	- High levels of homogentisic acid and its metabolites - Upregulation of phase II biotransformations - Treatment lowers the levels of homogentisic acid
Urea cycle disorders [32]	Human plasma	N/A	Standard of care	N/A	- Pyrimidine metabolism upregulated - Lower levels of BCAAs - Metabolic by-products of treatments found
Urea cycle disorders [36] (CPS1 deficiency)	Human plasma, urine, and breath	N/A	N-carbamyl-L-glutamate	1-^13^C-acetate	- Ureagenesis was low in patients, but increased upon treatment - Blood urea nitrogen increased after treatment - Lower levels of glutamine and alanine with treatment
Urea cycle disorders [37] (NAGS deficiency)	Human plasma, urine, and breath	N/A	N-carbamyl-L-glutamate	1-^13^C-acetate	- Ureagenesis was low in patients, but increased upon treatment - Blood urea nitrogen increased after treatment - Lower levels of glutamine and alanine with treatment
Disorders affecting molecule transport					
GLUT1 deficiency [28]	Human plasma, urine, and cerebrospinal fluid	N/A	Ketogenic diet	N/A	- Decreased carbohydrate metabolism - Biggest alterations in lipid metabolism - Altered amino acid catabolism - Very low levels of free L-carnitine
GLUT1 deficiency [45]	Mouse forebrains	Antisense-GLUT1 injection in embryos	N/A	N/A	- Low acetyl-CoA - Normal TCA cycle - Low levels of free fatty acids
Disorders affecting energy metabolism					
Fatty acid oxidation disorder [34] (VLCAD deficiency)	Human plasma	N/A	Standard of care vs. triheptanoin	N/A	- Repletion of TCA cycle intermediates better with triheptoanoin - Enrichment of very long-chain carnitine esters, no difference between two treatments
LIPT1 deficiency [62]	Human plasma and fibroblasts, mouse model	Transfection with wild-type LIPT1	N/A	U-^13^C-glutamine	- Higher glycolytic flux - More reductive TCA cycle compared to WT - Fatty acid synthesis mostly glutamine-dependent (instead of glucose-dependent) - Wild-type LIPT1 restored glutamine oxidation
EARS2 deficiency [63]	Human fibroblasts	N/A	N/A	N/A	- Decreased TCA cycle intermediates - Perturbations in amino acid, purine, and pyrimidine metabolism. - Higher levels of BCAAs and ophthalmate
Mitochondrial dysfunction [59] (SLC25A1 deficiency)	Human fibroblasts	N/A	N/A	U-^13^C-glucose	- Impaired mitochondrial citrate efflux causes - High levels of D2- and L2-hydroxyglutaric acid - Increased efflux of other TCA intermediates. Less citrate and isocitrate formation.
Mitochondrial dysfunction [60] (SLC25A1 deficiency)	Human fibroblasts	Transfection with wild-type SLC25A1	N/A	N/A	- Restored citrate efflux - Decreased levels of D2- and L2-hydroxyglutaric acid
Mitochondrial dysfunction [50] (Complex I, II, III, and CoQ)	*Caenorhabditis elegans*	Purchased with missense mutation	N/A	N/A	- Upregulation of OXPHOS, glycolysis, pyruvate metabolism, and the TCA cycle - Altered amino acid metabolism (Lower phenylalanine, arginine, higher BCAAs, alanine) - Cellular defense pathways depleted (cytochrome P450, glutathione)
Mitochondrial dysfunction [51] (Complex I, II, III, and CoQ, TCA cycle, pyruvate metabolism defect, nutrient-sensing)	*Caenorhabditis elegans*	Purchased with missense mutation	N/A	1,6-^13^C-glucose U-^13^C-glucose	- Reduced NAD+/NADH ratio - High glycolytic flux to lactate - Decreased TCA cycle activity
Mitochondrial dysfunction [49] (Propionic acidemia)	*Caenorhabditis elegans*	Purchased with missense mutation	N/A	1,6-^13^C_2_-glucose	- High glycolytic flux to lactate - Decreased flux of distal TCA cycle - Decreased cysteine levels, which is an important antioxidant
Mitochondrial dysfunction [35] (MELAS)	Human plasma	N/A	Arginine vs. citrulline supplementation	Yes	- Citrulline more efficient in restoring arginine and citrulline fluxes - More restoration of NO synthesis with citrulline - Normalized alanine and BCAA levels
Mitochondrial dysfunction [56] (Impaired mtDNA replication)	HEK-293-derived cells	Transfection with mutant DNA polymerase gamma to halt mtDNA replication	N/A	^13^C-glucose	- High levels of serine - Use more glucose to produce serine - Take up less serine from the medium - Impaired one-carbon metabolism
Mitochondrial dysfunction [57] (ATP synthase mutation)	Isogenic cell lines from female human osteosarcoma	Mitochondrially targeted zinc-finger nucleases, varying levels of mtDNA mutation	N/A	U-^13^C-glucose 4-^2^H-glucose U-^13^C-glutamine 1-^13^C-glutamine U-^13^C-aspartate	- Reduced NAD+/NADH ratio - Reductive carboxylation of glutamine - Glycolytic switch - Low levels of aspartate
Disorders affecting large molecules					
Glycogen storage disease [61] (GSD I and III)	Human fibroblasts	N/A	N/A	N/A	- Decreased activity of glycolysis - More expenditure of TCA metabolites into OXPHOS - Lower concentrations of total cysteine and glutathione
Glycogen storage disease [33] (GSD I)	Human plasma	N/A	N/A	N/A	- Increased de novo lipogenesis - Glycolytic switch - More use of glutamine
Glycogen storage disease [41] (GSD Ia)	Human plasma	N/A	N/A	1-^13^C-acetate	- Increased glycolytic flux - More fatty acid and cholesterol synthesis - High saturated fatty acid levels despite low levels in diet
Glycogen storage disease [46] (GSD Ia)	Mice, mouse hepatocytes	Mice expressing inducible CRE-ERT2 recombinase were treated with tamoxifen to delete exon 3 from G6pc	N/A	1-^13^C-galactose ^2^H-galactose	- Only 5% residual activity, but 30% endogenous glucose production - No endogenous glucose after inhibiting hepatic α-glucosidases
Lysosomal storage disease [38] (MPS I)	Human urine	N/A	N/A	N/A	- Elevated glycosaminoglycan levels - Dysregulation of amino acid catabolism (Arginine-proline metabolism is the most altered pathway) - High levels of aspartic acid - Alterations in urea cycle
Lysosomal storage disease [39] (MPS III)	Human urine	N/A	N/A	N/A	- Dysregulation of amino acid catabolism (Arginine-proline metabolism is the most altered pathway) - High levels of aspartic acid - Alterations in urea cycle and fatty acid metabolism
Lysosomal storage disease [40] (MPS VI)	Human urine	N/A	N/A	N/A	- Dysregulation of amino acid catabolism (Arginine-proline metabolism is the most altered pathway) - Large alterations in glutathione and histidine metabolism - Alterations in urea cycle and fatty acid metabolism
Lysosomal storage disease [43] (MPS IIIB)	Mouse plasma	Induced mutation within exon 6 by replacing fragment with cassette	rAAV gene delivery (enzyme replacement therapy)	N/A	- Amino acids, carbohydrates, vitamins, cofactors, lipids downregulated - Lower energy production - Treatment corrected 89–100% of affected metabolites
Congenital disorders of glycosylation [64] (PGM1-CDG)	Human fibroblasts	N/A	Galactose	U-^13^C-glucose U-^13^C- galactose	- Increased pentose phosphate pathway - Galactose treatment replenishes severely depleted galactose-1-phosphate levels and restores normal UDP-glucose and UDP-galactose levels - More use of exogenous galactose in N-glycans
Congenital disorders of glycosylation [65] (MPI-CDG)	Human fibroblasts	N/A	N/A	1,2-^13^C-glucose 4-^13^C-mannose	- N-glycans are built from exogenous glucose or mannose - No contribution of mannose from glycogen, gluconeogenesis, or recycling from broken down glycans
Congenital disorders of glycosylation [67] (TRAPPC9 deficiency)	Human fibroblasts	N/A	N/A	U-^13^C-glucose	- Increased upper glycolytic intermediates - Increased flux of glucose into TCA - Higher levels of pentose phosphate pathway activity - Higher UDP-hexose, lower sialic acid - Lower ratio of NAD+/NADH.
PGM2L1 deficiency [66]	Human fibroblasts	N/A	N/A	N/A	- Low levels of hexose- and pentose-bisphosphates - Normal levels of NDP-sugars - No indication of a glycosylation defect

BCAAs: branched-chain amino acids, ENU: N-ethyl-N-nitrosourea, MELAS: mitochondrial encephalomyopathy, lactic acidosis, and stroke-like episodes, MPS: mucopolysaccharidosis, mtDNA: mitochondrial DNA, N/A: not applicable, NO: nitric oxide, TCA cycle: tricarboxylic acid cycle.
